# Halophiles and Their Biomolecules: Recent Advances and Future Applications in Biomedicine

**DOI:** 10.3390/md18010033

**Published:** 2019-12-30

**Authors:** Paulina Corral, Mohammad A. Amoozegar, Antonio Ventosa

**Affiliations:** 1 Department of Biology, University of Naples Federico II, 80126 Naples, Italy; pcv@us.es; 2 Department of Microbiology and Parasitology, Faculty of Pharmacy, University of Sevilla, 41012 Sevilla, Spain; 3 Department of Microbiology, School of Biology, College of Science, University of Tehran, Tehran 14155-6955, Iran; amoozegar@ut.ac.ir

**Keywords:** halophilic bacteria, archaea and fungi, biomolecules, biomedicine, antimicrobial compounds, anticancer compounds

## Abstract

The organisms thriving under extreme conditions better than any other organism living on Earth, fascinate by their hostile growing parameters, physiological features, and their production of valuable bioactive metabolites. This is the case of microorganisms (bacteria, archaea, and fungi) that grow optimally at high salinities and are able to produce biomolecules of pharmaceutical interest for therapeutic applications. As along as the microbiota is being approached by massive sequencing, novel insights are revealing the environmental conditions on which the compounds are produced in the microbial community without more stress than sharing the same substratum with their peers, the salt. In this review are reported the molecules described and produced by halophilic microorganisms with a spectrum of action in vitro: antimicrobial and anticancer. The action mechanisms of these molecules, the urgent need to introduce alternative lead compounds and the current aspects on the exploitation and its limitations are discussed.

## 1. Halophilic Microorganisms

Halophiles are organisms represented by archaea, bacteria, and eukarya for which the main characteristic is their salinity requirement, halophilic “salt-loving”. Halophilic microorganisms constitute the natural microbial communities of hypersaline ecosystems, which are widely distributed around the world [1]. They require sodium ions for their growth and metabolism. Thus, based on the NaCl optimal requirement for growth the halophiles are classified in three different categories: slight (1–3%); moderate (3–15%); and extreme (15–30%) [2,3]. In contrast to halotolerant organisms, obligate halophiles require NaCl concentrations higher than 3% NaCl or above of seawater, with about 3.5% NaCl [4]. The tolerance parameters and salt requirements are dependent on temperature, pH, and growth medium. In this way, the halophiles are adapted and limited by specific environmental factors. Those microorganisms able to survive and optimally thrive under a wide spectrum of extreme environmental factors are designed polyextremophiles [5,6]. In fact, a halophilic microorganism can also be alkaliphile, designated as haloalkaliphile, growing optimally or very well at pH values above 9.0, but cannot grow at the near neutral pH value of 6.5 [7].

The general features of halophilic microorganisms are the low nutritional requirements and resistance to high concentrations of salt with the capacity to balance the osmotic pressure of the environment [8]. Their mechanisms of haloadaptation are based on the intracellular storage of KCl over 37% (5 M) (salt-in strategy) or the accumulation of compatible solutes (salt-out strategy) to keep the balance of sodium into the cytoplasm and counteract the osmotic pressure of the external environment given by the high salinity [9]. They are physiologically diverse; mostly aerobic and as well anaerobic, heterotrophic, phototrophic, and chemoautotrophic [10,11]. Ecologically, the halophilic microorganisms inhabit different ecosystems characterized by a salinity higher than seawater, i.e., 3.5% NaCl, these niches go from hypersaline soils, springs, salt lakes, sabkhas, and other naturally-occurring coastal saline habitats, marshes, marine abyssal sediments to endophytes [12]. Other known habitats are the result of human intervention like salted foods, brines, oil fields, saltern ponds and tanneries [13]. The high salinity reduces the number of organisms where just halophilic or halotolerant ones can survive in such hypersaline ecosystem, with archaea typically dominating the higher salinity environments. The predominant natural habitats better studied are the hypersaline lakes of oceanic (thalassohaline) or non-oceanic (athalassohaline) origin and solar salterns [14,15,16]. The better known hypersaline environments are the Great Salt Lake and the Dead Sea, with pH values around 7, and soda lakes with highly alkaline values of pH 9–11, among them are the Lake Magadi in Kenya, the Wadi Natrun lakes in Egypt, Mono Lake, Big Soda Lake, Soap Lake in Western USA, and Kulunda Steppe soda lakes in Russia [17]. Many new species of bacteria and archaea have been reported from various hypersaline regions located in different countries, mainly China, Spain, USA, Austria, Australia, Egypt, Korea, Japan, Iran, Thailand, Indonesia, Russia, Argentina, Kenya, Mexico, France, Poland, Philippines, Taiwan, Romania, and India [10,18,19]. The vast majority of halophilic bacteria and archaea produce carotenoid pigments, present in high amount in their membranes. The dense community of halophiles and the algae *Dunaliella*, also producer of carotenoids, are the responsible of the typical pink, red, and purple coloration of the hypersaline environments [20].

## 2. Biotechnological Importance/Interest of Haloarchaea and Halophilic Bacteria

The exploitation of extremophiles is having special importance in the development of new molecules with potential applications in biomedicine. Current efforts are focused primarily to cover the urgent health needs, especially those that represent the main global threats, cancer and antibiotic resistance. The great metabolic versatility of halophilic microorganisms, their low nutritional requirements and their genetic machineries of adaptation to harsh conditions, like nutrient starvation, desiccation, high sun radiation, and high ionic strength, make them promising candidates and a hope for drug discovery [21]. Continuous advances in “omics” and bioinformatic tools are revealing uncountable encoding genes for the production of several active compound in response to the extreme conditions [22,23]. The concomitant application of cutting-edge technologies is helping to deciphering the molecular, physiological, and metabolic mechanisms for the production of new bioactive compounds [24].

Halophilic microorganisms are recognized producers of carotenoid pigments, retinal proteins, hydrolytic enzymes, and compatible solutes as macromolecules stabilizers, biopolymers, and biofertilizers [19,25]. Halophilic bacteria and extremely halophilic aerobic archaea, also known as haloarchaea, play a significant role in the industry with a large number of applications like fermented food products, cosmetics, preservatives, manufacturing of bioplastics, photoelectric devices, artificial retinas, holograms, biosensors, etc. [26,27,28,29,30,31].

In this review, we focus on the biomolecules described as antimicrobial or anticancer compounds produced by halophilic bacteria, archaea, or fungi and discuss current and future perspectives in this field. 

## 3. Antimicrobial Compounds

The current situation of antibiotic resistance propagation poses a global threat to public health. Over the past decades, antibiotics have saved millions of lives, but their misuse has led to the emergence of multi-drug resistant bacteria (MDR), reducing or nullifying their effectiveness. Recently, the continuous increase in antibiotic resistance is reaching critical levels, which implies an increase in morbidity in the healthy population and an imminent risk for hospitalized patients [32,33]. In fact, the main cause of death of inpatients are attributable to complications due to MDR infections [34]. Preventing the return to the pre-antibiotic era is one of the main challenges for science. The urgent need to introduce new effective antimicrobial therapies is leading to the exploitation of all possible natural and sustainable resources, including extreme environments as a promising resource for new antibiotic discovery.

The first antimicrobial compounds from halophilic microorganisms were reported in 1982 by Rodriguez-Valera et al. Halocin was the term coined for substances secreted by several members of the genus *Halobacterium* capable of causing death and lysis of the surrounding microbiota. Halocins are the proteins and antimicrobial peptides (AMPs) produced by haloarchaea [35,36]. Despite the ecological and environmental role of several halocins, their action against human pathogens has been less studied.

In the fight against time, the clinical significance of halophilic microorganisms is minorly reported and the antimicrobial action against the most important risk group of human pathogens ESKAPE: *Enterococcus faecium*, *Staphylococcus aureus*, *Klebsiella pneumoniae*, *Acinetobacter baumannii*, and *Pseudomonas aeruginosa*, still remains as a potential.

According to the data inferred, the antagonistic action identified and the production of bioactive compounds by halophilic microorganisms are derived from bacteria, archaea, and fungi. In the chronology of AMPS discovery, several authors have gone beyond the primary screenings deciphering the chemical structure of the molecules in bacteria (Table 1), while the vast majority of inhibitory studies are solely limited to the activity (Table 2).

### 3.1. Bacteria

Members of the phylum *Actinobacteria* are mainly responsible for the inhibitory activity against human pathogens with clinical significance. As in non-extreme environments, in saline and hypersaline environments heterotrophic bacteria are also present in soils, being *Actinobacteria* frequently isolated from solar salterns, mangroves, and seafloor sediments [37,38]. The most frequent producers of metabolites reported come from species of the genus *Nocardiopsis* and *Streptomyces*, hence constituting the main producers of bioactive compounds. In fact, members of the genus *Streptomyces* are widely recognized as fruitful producers of natural compounds [39]. The chemical elucidation of molecules known from halophilic members of *Nocardiopsis* are: (i) pyrrolo (1,2-A (pyrazine-1,4-dione, hexahydro-3-[2-methylpropyl]-) and Actinomycin C2, two compounds produced by the haloalkaliphilic strain *Nocardiopsis* sp. AJ1, isolated from saline soil of Kovalam solar salterns in India [40]; (ii) Angucyclines and Angucyclinones are produced by *Nocardiopsis* sp. HR-4, isolated from a salt lake soil in Algerian Sahara, the new natural compound was established as 7-deoxy-8-O-methyltetrangomycin, which is also effective against Methicillin-Resistant *Staphylococcus aureus* (MRSA) ATCC 43300 [41]; (iii) Borrelidin C and D are produced by *Nocardiopsis* sp. HYJ128, isolated from topsoil saltern in Jeungdo, Jeollanamdo, Republic of Korea, exhibited antimicrobial action against *Salmonella enterica* ATCC 14028 [42]; (iv) Quinoline alkaloid (4-oxo-1,4-dihydroquinoline-3-carboxamide) was identified as a new natural product from *Nocardiopsis terrae* YIM 90022 isolated from saline soils in China. The antibacterial activity of the quinolone was reported in *S. aureus*, *B. subtilis* and *E. coli*; the quinolone has also antifungal activity against the pathogenic fungi, as it was observed against *Pyricularia oryzae*. Another five known compounds were also produced by *N. terrae* YIM 90022 [43]; (v) new p-terphenyls: p-terphenyl 1 and a novel p-terphenyl derivative bearing a benzothiazole moiety are produced by halophilic actinomycete *Nocardiopsis gilva* YIM 90087, isolated from a hypersaline soil Xinjiang, China. Furthermore, of the antimicrobial activity against clinical strains, these compounds exhibit antifungal activity against species of *Fusarium, Trichophyton, Aspergillus, Candida,* and *Pyricularia.* Known molecules like p-terphenyl 2, novobiocin, cyclodipeptides, and aromatic acids are also produced by *N. gilva* YIM 90087, which is considered as a new source for novobiocin [44].

Regarding the metabolites produced by members of the genus *Streptomyces*, only a low number of strains has been isolated from hypersaline environments; however, members of this genus are frequently isolated from marine deep or coastal sediments where the salinity is higher than that of seawater. Among the molecules identified are: (i) 1-hydroxy-1-norresistomycin, this quinone-related antibiotic was extracted from *Streptomyces chibaensis* AUBN1/7, isolated from marine sediment samples of the Bay of Bengal, India. This compound exhibited antibacterial activities against Gram-positive and Gram-negative bacteria, besides of a potent in vitro cytotoxic activity against cell lines HMO2 (gastric adenocarcinoma) and HePG2 (hepatic carcinoma) [45]; (ii) Himalomycin A and Himalomycin B, two new anthracycline antibiotics produced by *Streptomyces* sp. strain B692, isolated from sandy sediment of a coastal site of Mauritius (Indian Ocean). In addition, known metabolites like rabelomycin, fridamycin D, N benzylacetamide, and N-(2′-phenylethyl) acetamide were also produced by *Streptomyces* sp. strain B692 [46]; (iii) 7-demethoxy rapamycin was produced by a moderately halophilic strain *Streptomyces hygroscopicus* BDUS 49, isolated from seashore of Bigeum Island, South West coast of South Korea; the molecule displayed a broad spectrum antimicrobial activity against Gram-positive and Gram-negative bacteria. Antifungal and cytotoxic action was also identified on this strain [47]; (iv) Streptomonomicin (STM) is an antibiotic lasso peptide from *Streptomonospora alba* YIM 90003, isolated from a soil sample in Xinjiang province, China. STM is active against several Gram-positive bacteria, in particular species of *Bacillus, Listeria, Enterococcus, Mycobacterium* and *Staphylococcus*. Despite that STM has an inhibitory action against a wide panel of Gram-positive pathogens, the activity against fungi and Gram-negative bacteria was not evidenced [48].

In addition to the mentioned genera of *Actinobacteria* (*Nocardiopsis* and *Streptomyces*), recognized as the more prolific producers of natural substances, other halophilic species belonging to different genera have also been described as producers of molecules like: (i) cyclic antimicrobial lipopeptides: Gramicidin S and four cyclic dipeptides (CDPs), named cyclo(l-4-OH-Pro-l-Leu), cyclo(l-Tyr-l-Pro), cyclo(l-Phe-l-Pro), and cyclo(l-Leu-l-Pro), were extracted from *Paludifilum halophilum* strain SMBg3, which constitute a new genus of the family *Thermoactinomycetaceae*, isolated from superficial sediment collected from Sfax marine solar saltern in Tunisia. These CDPs possess an inhibitory effect against the plant pathogen *Agrobacterium tumefaciens* and the human pathogens *Staphylococcus aureus, Salmonella enterica, Escherichia coli,* and *Pseudomonas aeruginosa* [49]; (ii) A semi synthetic derivative N-(4-aminocyclooctyl)-3,5-dinitrobenzamide, obtained from the precursor of the novel natural product cyclooctane-1,4-diamine and a known compound 3-([1H-indol-6-yl] methyl) hexahydropyrrolo [1,2-a] pyrazine-1,4-dione were obtained from *Pseudonocardia endophytica* VUK-10, isolated from sediment of Nizampatnam mangrove ecosystem in Bay of Bengal, India. The new compound, semi synthetic derivative N-(4-aminocyclooctyl)-3,5-dinitrobenzamide showed a strong antimicrobial and antifungal activity against *Streptococcus mutans*, *Pseudomonas aeruginosa, Candida albicans,* and *Aspergillus niger*. Significant anticancer activities at nanomolar concentrations were also observed in carcinoma cell lines MDA-MB-231 (breast), HeLa (cervical), OAW-42 (ovarian), and MCF-7 (breast) reported as resistant to cancer drugs [50]. In minor grade, other halophilic bacteria not belonging to the phylum *Actinobacteria* produce antimicrobial compounds, as for example halophilic strains of the genus *Vibrio*, like *Vibrio* sp. A1SM3-36-8, isolated from Colombian solar salterns, which produces 13-cis-docosenamide with special antimicrobial action against Methicillin-resistant *Staphylococcus aureus* (MRSA) and cytotoxic activity against cervical adenocarcinoma (SiHa) and lung carcinoma (A-549) [51]. Within this genus, *Vibrio parahaemolyticus* strain B2 is recognized by producing Vibrindole A, and was also effective against *Staphylococcus aureus* [52].

Finally, *Bacillus* sp. BS3 [53] and *Halomonas salifodinae* MPM-TC [54] showed antimicrobial action against *Pseudomonas aeruginosa*. Both strains were isolated from solar salterns in Thamaraikulam, Tamil Nadu, India. In the case of *Halomonas salifodinae* MPM-TC, besides of the inhibition of bacterial growth also exhibits an antiviral action against the White Spot Syndrome Virus (WSSV) in the white shrimp *Fenneropenaeus indicus*. The effect suppressor of the virus and the boosting of immune system of the shrimps make of the extracted compound a feasible alternative to commercially banned antibiotics and excellent candidate to develop new antiviral drugs against shrimp viruses such as WSSV.

A genome-mining study conducted on 2699 genomes across the three domains of life demonstrated the widespread distribution of non-ribosomal peptide synthetase (NRPSs) and modular polyketide synthase (PKSs) biosynthetic pathways. Among 31 phyla of bacteria inferred, *Actinobacteria* is the most representative exhibiting the presence of 1225 gene clusters between NRPS, PKS and hybrids from a total of the 271 genomes studied. It was observed that *Salinispora arenicola* CNS-205 and *Salinispora tropica* CNB-440 harbor PKS and NRPS gene clusters, respectively. The halophilic bacterium *Halomonas elongata* DSM 2581 also contains NPRS [55].

The biotechnological potential of halophilic bacteria, especially for antimicrobial exploitation, still remains in progress, in spite that the occurrence of new several groups of microorganisms is high, the rate of discovery of new biomolecules is low compared with non-halophilic bacteria. Despite periodic descriptions of new species and attempts to culture hidden microbiota, there are no significant studies focused on the discovery of new bioactive metabolites produced by microorganisms from hypersaline ecosystems. The genome-guided studies are currently the best support to take novel strategies in drug discovery. All the antimicrobial compounds described herein derived from halophilic bacteria in which the molecule has been elucidated are summarized in Table 1 and the strains capable of inhibiting pathogens in primary tests whose molecules are unknown are shown in Table 2.

### 3.2. Archaea

Since the discovery of halocins and their action against the surrounding microbiota in their habitats [35] no new or known antimicrobial compounds derived from archaea capable of inhibiting human pathogens have been reported in the literature to date. At an ecological level, the role of archaeocins in microbial communities is the interspecies competition, the antimicrobial activity of halocins suggests that its function is to dominate a given niche occupied by microorganisms having similar adaptations and nutritional requirements [83,84,85]. Members of *Halorubrum* and *Haloferax* have been identified as the preponderant halocin-producing genera, the cross-domanin antimicrobial action was observed against bacterial members of the genera *Halomonas*, *Rhodovibrio*, *Salisaeta*, or *Pontibacillus*, all isolated from hypersaline samples [86].

To understand the current situation, it is necessary that a comprehensive analysis of the possible reasons why haloarchaea are under-explored at the biotechnological level and why the antimicrobial exploitation is scarce in comparison with other microorganisms prevenient of non-halophilic environments. The first limitation found is the cultivation time of haloarchaea, observed at around 5 to 30 days to yield colonies or cellular density in broth cultures [12]. Once the cultivation is reached, the upcoming drawback is the evaluation of the inhibitory capacity of haloarchaea against a panel of human pathogens. The main obstacle to overcome is when the primary screening (isolate vs. pathogen) is performed due to the high salinity requirements of haloarchaea to grow, greater than 20% of NaCl until saturation, while in halophilic bacteria the screening can be adapted at lower range of salinity, under 15% of NaCl.

Tests such a direct spot-inoculation of the supernatant, diffusion discs, and cross-streak require the adaptation of an appropriate protocol. Finding the same and suitable conditions to test both microorganisms drive to set-up alternative technical procedures, like dual-media and crude extracts for testing those strains growing above the seawater salinity, ca. 3.5 % [87]. Another possible reason is that the study of extremophilic microbiota has been approached at an ecological level and the vast biotechnological exploitation of these extremophiles is more recognized on their enzymes and compatible solutes. The low metabolic requirements, the hypersaline conditions where they thrive, or the low competition for nutrients with their peers determine their behavior, i.e., the production of halocins, which action is limited to the closest members inhabiting in the same environment [88,89]. This could explain that the production of antimicrobials against the non-halophilic community of microorganisms seems to be unnecessary.

Constituted as a powerful tool, “omics” approaches as metagenomics and genomics effectively support ecological and bioprospecting studies deciphering new insights into halophilic microorganisms [90,91,92]. Extremely rare is the interdomain horizontal gene transfer (IHGT) across bacteria, archaea, and fungi of homologous DNA. However, a genomic-guided study revealed for the first time a potent antibacterial gene encoding a glycosyl hydrolase 25 muramidases (GH25-muramidase) identified in archaea after co-cultivation with a bacterial competitor [93]. In the genome-mining study conducted by Wang et al. (2014), an atlas of nonribosomal peptide synthetase (NRPSs) and modular polyketide synthase (PKSs) gene clusters was built based on 2699 genomes of bacteria, archaea, and fungi. In this study, were included 25 members of *Halobacteria: Haloarcula hispanica* ATCC 33960, *Halalkalicoccus jeotgali* B3, *Haloarcula marismortui* ATCC 43049, *Halobacterium* sp. NRC-1, *Halobacterium salinarum* R1, *Haloferax mediterranei* ATCC 33500, *Haloferax volcanii* DS2, *Halogeometricum borinquense* DSM 11551, *Halomicrobium mukohataei* DSM 12286, *Halopiger xanaduensis* SH-6, *Haloquadratum walsbyi* C23, *Haloquadratum walsbyi* DSM 16790, *Halorhabdus tiamatea* SARL4B, *Halorhabdus utahensis* DSM 12940, *Halorubrum lacusprofundi* ATCC 49239, *Haloterrigena turkmenica* DSM 5511, *Halovivax ruber* XH-70, *Natrialba magadii* ATCC 43099, *Natrinema* sp. J7-2, *Natrinema pellirubrum* DSM 15624, *Natronobacterium gregoryi* SP2, *Natronococcus occultus* SP4, *Natronomonas moolapensis* 8.8.11, *Natronomonas pharaonis* DSM 2160, *Salinarchaeum* sp. Harcht-Bsk1. Of a total of 3339 cataloged gene clusters, no PKS, NPKS or hybrid in *Halobacteria* were reported. Within the studied archaea, only two and one NRPS were identified in *Methanobacteria* and *Methanomicrobia*, respectively [55]. Despite these results and considering that the class *Halobacteria* is wide represented with seven families, these results do not exclude the biosynthetic capacity of nonribosomal peptide and polyketide, and nor discourage the biotechnological interest of haloarchaea for future natural product discovery.

### 3.3. Fungi

Along the years of research on natural products, fungi represent the basis of antimicrobial discovery. Halotolerant and halophilic fungal communities that inhabit the natural hypersaline environments are not strictly salt requiring, as they can grow and adjust to the whole salinity range, from freshwater to almost saturated NaCl solutions [94,95]. Despite this versatility, the vast majority of antimicrobial molecules from halophilic fungi have been produced under low or moderate salinity conditions since the primary screenings against SKAPE microorganisms are easier without NaCl. The mycobiota of hypersaline environments is dominated by members of *Aspergillus*, *Penicillium*, and other genera, such as *Alternaria*, *Cladosporium*, *Fusarium*, *Debaryomyces*, *Scopulariopsis*, *Chaetomium*, *Wallemia*, and *Hortaea*, which are well represented in ecological and biodiversity studies [96,97]. The species *Gymnoascus halophilus*, *Aspergillus penicillioides*, *Hortaea werneckii*, *Phaeotheca triangularis*, *Aureobasidium pullulans*, *Trimmatostroma salinum*, and some species of the genus *Wallemia*, like *W. ichthyophaga*, are recognized as obligately halophilic, or require high levels of salt above that of seawater [98,99]. However, antimicrobial compounds have not been reported from these species.

The halophilic species of the genus *Aspergillus* are the most prolific and several strains of *Aspergillus* sp. have been isolated from Arctic sub-sea sediments from the Barents Sea (Table 3). In particular, strain 8Na identified as *A. protuberus*, a polyextremophilic fungus able to grow in a wide range of pH, temperature and salinity (up to 25% (*w/v*)) showed an antimicrobial efficacy against human pathogens. The strongest power inhibitory action was observed against *Staphylococcus aureus*. The molecule responsible of the activity was identified as Bisvertinolone, a compound member of the family Sorbicillinoid [87]. *Aspergillus flocculosus* PT05-1 and *Aspergillus terreus* PT06-2, both isolated from sediment of Putian sea saltern of Fujian, China, showed antimicrobial activity against *Enterobacter aerogenes*, *Pseudomonas aeruginosa*, and *Candida albicans.* Strain PT05-1 produces 11 metabolites among which two are new ergosteroids and pyrrole derivative compounds [100], and strain PT06-2 produces the novel compounds: Terrelactone A and Terremides A and B [101]. Other strains of the genus *Aspergillus*, like *A. terreus* Tsp22 [101,102,103], *A. flavus*, *A. gracilis*, and *A. penicillioids* [102] have antibacterial and antioxidant activities in crude extracts but the molecule has not been identified. In the atlas of Wang et al. (2014), 360 fungi were genome-mined cataloguing a total of 307 gene clusters from 30 strains of the phylum *Ascomycota*. Within this group, strains of the genus *Aspergillus: A. nidulans* FGSC A4, *A. fumigatus*, *A. niger* CBS 513 88, and *A. oryzae* RIB40 harbor NRPSs, PKSs and hybrids gene clusters [55]. These results confirm that the genus *Aspergillus* is among the most prolific producers of antimicrobial metabolites. In spite of the prosperous production of compounds from fungi, the active molecules derived from extremely halophilic fungi are still scarce (Table 3). It is highly probable that through genome-driven studies in halophilic fungi, NRPSs and PKSs are substantially present as their peers providing new insights into the fungal biosynthetic pathways.

## 4. Anticancer Compounds

Natural products are relevant anticancer drugs, which are also called bioactive molecules, produced by organisms. Although, earlier and the well-established anticancer natural products have been obtained from plant cells originally, microorganisms are an excellent alternative, due to the diversity of the microbial world, their easy manipulation, and they can be screened physiologically to discover new natural products with antitumor activity. Although bacterial cells have different communication methods with tumor cells other than metabolites experimentally, bacterial metabolites have been considered the most conventional way against cancer cells viability. Today, more attention is focused on extremophiles as a new source of novel biomolecules [104,105]. Among extremophiles, halophilic and halotolerant microorganisms, which inhabit hypersaline environments, are considered as reliable sources of antitumor metabolites with fewer side effects. In recent years, several studies have been focused on the importance of metabolites from halophilic microorganisms on cancer treatment. The halophilic bacteria, archaea, and fungi involved on the production of anti-cancer biomolecules are summarized in Table 4.

### 4.1. Bacteria

Since the last two decades, halophilic bacteria have attracted the interests of researchers due to their adaptability to a wide range of salinities. Some studies have been carried out to determine the role of halophilic bacteria in cancer treatment. In one of these studies, Chen et al. (2010) assayed fourteen crude extracts from 45 halophilic bacterial strains and showed cytotoxic activity against human liver cancer cell line Bel 7402 with a half maximal inhibitory concentration (IC_50_) of 500 μg/mL and five of them showed remarkable activities with IC50 lower than 40 μg/mL [106]. The antineoplastic antibiotic known as tubercidin, was isolated from the halophilic actinobacterium *Actinopolyspora erythraea* YIM 90600, this compound exhibited the capability to stabilize the tumor suppressor Programmed Cell Death Protein 4 (Pdcd4), which is known to antagonize critical events in oncogenic pathways. Tubercidin, significantly inhibited proteasomal degradation of a model Pdcd4-luciferase fusion protein, with an IC_50_ of 0.88 ± 0.09 μM, unveiling a novel biological activity for this well-studied natural product [107].

In two studies on different extracts of halophilic and halotolerant bacteria isolated from brine-seawater interface of the Red Sea, Sagar et al. (2013) tested the cytotoxic and apoptotic activity of their extracts against three human cancer cell lines, including HeLa (cervical carcinoma), MCF-7 (breast adenocarcinoma) and DU145 (prostate carcinoma). In one of their studies, a total of 20 lipophilic (chloroform) and hydrophilic (70% ethanol) extracts from twelve different strains were assessed. Among these, twelve extracts were found to be very active after 24 h of treatment, which were further evaluated for their cytotoxic and apoptotic effects at 48 h. The extracts from the isolates *Halomonas* sp. P1-37B, *Halomonas* sp. P3-37A, and *Sulfitobacter* sp. P1-17B were found to be the most potent against tested cancer cell lines [108]. In the other study, ethyl acetate extracts of 24 strains were assayed and the results showed that most extracts were cytotoxic against one or more cancer cell lines. Out of the thirteen most active microbial extracts, six extracts induced significantly higher apoptosis (>70%) in cancer cells. Molecular studies revealed that extracts from *Chromohalobacter salexigens* strains P3-86A and P3-86B followed the sequence of events of apoptotic pathway involving matrix metalloproteinases (MMP) disruption, Caspase-3/7 activity, Caspase-8 cleavage, polymeric adenosine diphosphate ribose polymerase 1 (PARP-1) cleavage, and phosphatidylserine exposure, whereas the extracts from another *Chromohalobacter salexigens* strain K30 induced Caspase-9 mediated apoptosis. The extracts from *Halomonas meridiana* strain P3-37B and *Idiomarina loihiensis* strain P3-37C were unable to induce any change in MMP in HeLa cancer cells and thus suggested a mitochondria-independent apoptosis induction. However, further detection of a PARP-1 cleavage product and the observed changes in Caspase-8 and Caspase-9 suggested the involvement of caspase-mediated apoptotic pathways [109]. An ethyl acetate extract from *Streptomyces* sp. WH26 showed significant cellular toxicity. Two new compounds, 8-*O*-methyltetrangulol and naphthomycin A, were isolated from this extract via silica gel column chromatography and high-pressure liquid chromatography (HPLC). These two compounds showed potent cytotoxic activity against several human cancer cell lines including A549, HeLa, BEL-7402, and HT-29 [110]. Novel anticancer molecules, Salternamide A–D, were isolated from a halophilic *Streptomyces* sp. isolated from a saltern on Shinui Island, in the Republic of Korea, and exhibited an extensive viability reduction in several cancer cell lines [111]. Among these molecules, Salternamide A inhibited the hypoxia-induced accumulation of HIF-1α in several cancer cell lines and suppressed the HIF-1α by downregulation of its upstream signaling pathways such as PI3K/Akt/mTOR, p42/p44 MAPK, and STAT3. Moreover, in human colorectal cancer cell lines, salternamide A caused cell death by arresting the cells in the G2/M phase and lead to apoptosis [112]. A halophilic bacterium, *Vibrio* sp. strain A1SM3-36-8, isolated from Manaure solar saltern in Colombia, showed a high potential to inhibit methicillin-resistant *Staphylococcus aureus* and causing a slight inhibition of lung cancer cell lines [51]. In another study, among nine moderately halophilic bacteria isolated from saline environments of Iran, the supernatant of four strains showed ability to reduce the viability of HUVEC cancer cell line while one of these supernatants induced the proliferation of adipose-derived mesenchymal stem cells [113]. The actinobacterium *Nocardiopsis lucentensis* DSM 44048 isolated from Salt marsh soil in Alicante, Spain produces a new benzoxazole derivatives, Nocarbenzoxazole G. The compound showed cytotoxic activity against liver carcinoma cells (HepG2) and HeLa cancer cells with IC50 values of 3 and 1 μM, respectively [114]. A halotolerant *Bacillus* sp. KCB14S006, which was isolated from a saltern, produced three new lipopeptides with cytotoxic activity. These new lipopeptides lead to a ~30% decrease in the viability of HeLa and src(ts)-NRK cells [115]. In another study, the methanolic extracts of *Bacillus* sp. VITPS14 and *Bacillus* sp. VITPS16 showed cytotoxicity against HeLa cancer cell line but not against A549 cells. These halophilic strains were isolated from soil samples of Marakkanam saltern and Pichavaram mangrove forest, India, respectively. Another halophilic strain, *Bacillus* sp. VITPS7, isolated from this area showed significant antioxidant activity. The presence of β-carotene and flavonoids was confirmed in these extracts [116]. In another study, twenty-four novel halophilic bacteria isolated from the surrounding of active volcanic Barren Island Andaman and the Nicobar Islands in India were examined for their cytotoxic activity against MDA-MB-231 breast cancer cell line. About 65% of these bacterial strains decreased the viability of this cell line to 50% or lower [117]. Metabolites from *Piscibacillus* sp. C12A1 isolated from Sambhar Lake, India, decreased the viability of MDA-MB-231 breast cancer cell line with downregulation of Bcl-xL and CDK-2 expression. Furthermore, cell migration and colony formation of the cells were inhibited in the presence of these metabolites [118]. 

Biosurfactants produced by microorganisms are active molecules that create an amphipathic surface containing hydrophilic and hydrophobic moieties. In recent years, these biomolecules were also found to possess several interesting properties of therapeutic and biomedical importance. Biosurfactants from the halophilic bacteria *Bacillus* sp. BS3 and *Halomonas* sp. BS4 had the ability to reduce the viability of mammary epithelial carcinoma cells to 24.8% and to 46.8 significantly (*p* < 0.05) at 0.25 μg/mL and 2.5 μg/mL concentrations, respectively [53,119].

Extracellular polymeric substances (EPS) have recently been attracting considerable attention because of their potential applications in many fields, including biomedicine. EPSs are heterogeneous polymers that contain a wide range of homo- or hetero-carbohydrates as well as organic and inorganic substituents. EPSs produced by both halophilic bacteria and archaea showed remarkable anticancer activity. Also, these polysaccharide polymers have been introduced as important agents for developing nanocarrier systems for anti-cancer drugs. For example, in 2011, Ruiz-Ruiz et al. showed that at a concentration of 500 μg/mL, the over sulfated exopolysaccharide of the halophilic bacterium *Halomonas stenophila* strain B100 completely blocked the proliferation of the human T leukemia cells (Jurkat cells) in a dose-response manner. Also, they revealed the positive effect of sulfate groups in viability reduction of Jurkat cells [120]. Moreover, in another study, the anti-cancer activity of the polysaccharide levan and its aldehyde-activated derivatives was reported. This polysaccharide was isolated from *Halomonas smyrnensis* AAD6 and its anticancer activity against human cancer cell lines such as lung (A549), liver (HepG2/C3A), gastric (AGS), and breast (MCF-7) cancer cells (Table 4) has been investigated. In this study, all evaluated cells were treated with levan samples at a broad concentration ranging from 10 to 1000 μg/mL. All samples were found to display growth inhibition against cancer cell lines at the highest dose (1000 μg/mL). Unmodified levan showed higher anti-cancer effect against AGS cells against other cancer cell lines. Aldehyde-activated levan showed higher anti-tumor activity than unmodified levan against all cancer cell lines. Oxidized levan samples showed higher anticancer activity against A549 and HepG2/C3A cells. By increasing the oxidation degree, the anti-cancer activity also increased. Therefore, it was clearly demonstrated that the introduction of the chemically modified group, aldehydes, into the linear levan molecule could significantly enhance the antitumor activity of levan polysaccharide [121]. 

Recent preclinical and medicinal studies have shown an inverse relationship between dietary uptake of carotenoids and cancer occurrence. It was reported that the extracted carotenoid from the halotolerant bacterium *Kocuria* sp. QWT-12, isolated from industrial tannery wastewater in Qom, in Iran, had the ability to reduce the viability of human breast cancer cell lines MCF-7, MDA-MB-468, and MDA-MB-231 with an IC50 of 1, 4, and 8 mg/mL, respectively. Also, this carotenoid decreased the viability of human lung cancer cell line A549, with IC50 of 4 mg/mL. This carotenoid did not reduce the viability of normal fibroblast cell line at these concentrations [122].

Among all anticancer enzymes, l-asparaginase and l-glutaminase are enzymes with the ability to inhibit acute lymphoblastic leukemia and other cancer cells. Halophilic and halotolerant bacteria are novel sources of these anticancer enzymes. For example, a screening from 85 halophilic strains from the hypersaline Urmia Lake in Iran revealed that 16 (19%) and three strains (3.5%) showed l-asparaginase and l-glutaminase activity, respectively. It was shown that l-asparaginase was produced mainly by strains belonging to the genus *Bacillus*, while l-glutaminase was produced mainly by strains of the genus *Salicola* [27]. In another study, it was reported that from 110 halophilic strains isolated from different saline environments of Iran, a total of 29, four, and two strains produced anticancer enzymes including l-asparaginase, l-glutaminase, and l-arginase, respectively. These strains belonged to the genera *Bacillus*, *Dietzia*, *Halobacillus*, *Rhodococcus*, *Paenibacillus*, and *Planococcus*, as Gram-positive bacteria, and *Pseudomonas*, *Marinobacter*, *Halomonas*, *Idiomarina*, *Vibrio*, and *Stappia* as Gram-negative bacteria [123]. From these strains, the anti-cancer activity of a novel recombinant l-asparaginase enzyme produced by *Halomonas elongata* strain IBRC M10216 was assayed against human lymphoblastic and myeloid leukemia cell lines, Jurkat and U937 (Table 4). This enzyme enhanced the viability of these cancer cell lines with IC50 values of 2 and 1 U/mL, respectively, but at these concentrations had no effect on the viability of normal HUVEC cell line [124].

### 4.2. Archaea

Although most studies in this field have been focused on halophilic bacteria, some studies investigated the potentials of haloarchaea. In one of these studies, among nine haloarchaeal strains isolated from Aran-Bidgol Salt Lake, in Iran, supernatant metabolites from *Halobacterium salinarum* IBRC M10715 had the most potent cytotoxic effect on prostate cancer cell lines (DU145 and PC3, IC50 = 0.5 mg/mL) without any effects on normal fibroblast cells (HFF-5). Moreover, the selective metabolite significantly increased both early and late apoptosis (about 11% and 9%, respectively) in the androgen-dependent PC3 cell line and reduced sphere formation ability of both cancer cell lines with down-regulation of SOX2 gene expression. Furthermore, prostate cancer cell tumors developed in nude mice significantly shrank post intratumor injection of the metabolite from *Halobacterium salinarum* IBRC M10715 [105]. *Halorubrum* sp. TBZ112 is a haloarchaeal species isolated from the Urmia Lake, Iran. It was reported that this strain could produce EPSs. The isolated EPSs possess a relatively low molecular weight in comparison with those EPSs isolated from other extreme environments (5 vs. ≥100 kDa, respectively) and the absence of sulfate functional groups in their structure was reported. The anticancer activity of the EPSs from *Halorubrum* sp. TBZ112 was examined and the results did not show any significant changes in the viability of gastric cancer cells (MKN-45) and normal human dermal fibroblast cells (HDF) at concentrations of 100, 250, 500, and 1000 µg/mL after 24 and 48 h of treatment. As the existence of sulfate functional groups and the EPSs bioactivities are directly related, the low cytotoxicity potential of the EPSs from *Halorubrum* sp. TBZ112 was not unexpected [125].

Both in vivo and in vitro studies confirm chemoprevention effects of some carotenoids anticancer activity. Halophilic microorganisms showed great potential toward the production of various carotenoids such as β-carotene, bacterioruberin, and xanthophylls. In recent years, some investigations were carried out to determine the role of carotenoids or other bioactive molecules produced by halophiles on cancer treatment. The effects of *Halobacterium halobium* carotenoid extract on the viability of human hepatoma, HepG2, have been analyzed. This haloarchaeal strain was isolated from a Tunisian solar saltern and the results emphasized that increasing concentrations of the carotenoid extract of this halophilic archaeon decreased significantly the viability of the HepG2 cancer cell line [126]. Carotenoids from the haloarchaea *Halogeometricum limi* strain RO1-6 and *Haloplanus vescus* strain RO5-8 showed a potent antioxidant activity in comparison with β-carotene. In addition, these carotenoid extracts inhibited HepG2 cells in vitro, in a dose-dependent manner. Bacterioruberin was the predominant carotenoid extracted from these haloarchaea [127].

### 4.3. Fungi

The biotechnological applications of halophilic fungi are remarkedly less studied in comparison with halophilic bacteria. There is only one study focused on the cytotoxic effect of metabolites from a moderately halophilic fungal strain, *Aspergillus* sp. F1 [128]. Based on this publication, this strain produced three compounds with anticancer activity including cytochalasin E, ergosterol, and rosellichalasin, and higher salt concentrations increased the production of these compounds. All isolated compounds decreased the viability of A549, Hela, BEL-7402, and RKO human cancer cell lines and the inhibition effect of ergosterol on human colon cancer cell line, RKO, was the most potent cytotoxic report in this study.

Table 4 summarize all the mentioned reports in Section 4, which are related to the anticancer effect of halophilic bacteria, archaea, and fungi isolated from different saline and hypersaline environments in the world.

The following table (Table 5) gathers the most promising new compounds derived from halophilic microorganisms. The minimum inhibitory concentration (MIC) and the half maximal inhibitory concentration (IC_50_) are shown, based on their in vitro bioactivity. The results suggest that these compounds could be candidates for preclinical trials.

## 5. Future Perspectives

As the prevalence of antimicrobial resistance increases, researchers are developing new technologies and strategies to find alternatives that reduce the morbidity and mortality caused by the MDR bacteria. Categorizing the need for obtaining new molecules, the most requested by the public health are antimicrobial and anticancer compounds according to the data annually reported by the World Health Organization (WHO). The current and future of natural product discovery is the application of a combination of multi-omics approaches. Depending on the phase of the study, it is foreseen genomics, metagenomics, transcriptomics, proteomics, and metabolomics to reveal the biosynthetic capabilities of a single microorganism or microbial communities in hypersaline environments. 

The discovery of novel lead compounds requires more that in silico predicted genes and large promising data. The current problem with massive approaches is precisely the lack of concrete results traduced in novel lead compound derived of “meta-omics” studies. The heterologous expression of biosynthetic genes is the bottleneck since in several cases the recombinant product and its expression is totally different from what was expected. However, it is important to emphasize that the cultivation of hidden and uncultivable microbiota is improving with the assessment of metagenomic studies [129,130].

Genome mining has been implemented as a mandatory tool widely used to characterize the genetic basis of secondary metabolite biosynthesis based on the features of secondary metabolites organized as biosynthetic gene clusters (BGCs), especially the profile of gene encoding key signature enzymes [131,132,133]. The application of Next Generation Sequencing (NGS) allows the study of microbial diversity every day more accessible and affordable that allows the prediction of cryptic metabolic pathways and genes involved in the activity. The genome-guided discovery relies on sophisticated methods for identification of knew gene families related clusters. The accurate prediction and analysis of relevant genes for secondary metabolite biosynthetic pathways in microbes is performed through the tool based on the Antibiotics and Secondary Metabolites Analysis Shell (antiSMASH) [134].

Due to the high rate of rediscovery of known compounds, the dereplication is an essential approach that allows the identification of duplicate molecules. Dereplication is relying on finding a matching of mass spectra with those present in the mass spectrometry data repository. The development of new computational tools like the algorithm searching spectral, DEREPLICATOR+ is helping to identifying in one order of magnitude peptidic natural products (PNPs) that include nonribosomal peptides (NRPs), and ribosomally synthesized and post-translationally modified peptides (RiPPs). The matching is extended to the identification of polyketides, terpenes, benzenoids, alkaloids, flavonoids, and other classes of natural products. One of the utilities of DEREPLICATOR+ is the enabling of cross-validation of genome-mining and peptidogenomics/glycogenomics results [135].

Several laboratories working in microbial bioprospecting keep their private collection once the antimicrobial, anticancer, antifungal, etc. activity is detected. In many cases, these positive isolates derived from primary screenings are not further studied by genome sequencing and dereplication. A common issue is the obtaining of the purified active compound under laboratory conditions with limited facilities and handling large data with a proper analysis. Moreover, it is important to consider the dereplication costs and time-consuming interpreting. The mentioned facts delay the biodiscovery attempts and constitute the reasonable causing of keeping a stored library of potential compounds. The projection of drug discovery product research is the simplification and accessibility to all these tools faster and with less effort. The power of genome mining in studying natural product biosynthesis by showing the widespread distribution of NRPS/PKS gene clusters and by the elicitation of previously unidentified pathways has been demonstrated. It is clear that coupling genome mining and dereplication will accelerate the biodiscovery at initial steps. The integration and linking of computational approaches are certainly the future of natural product research.

In this review, we have focused in all anticancer molecules reported from halophilic microorganisms. According to the cellular lines used, the focus of primary screenings is addressed to the leading cancer types that affect the global population. However, it is important that further screenings should include cellular lines with intrinsic chemoresistance, like sarcoma and glioblastoma, characterized by aggressive overproliferation. The future of novel anticancer agents seems to be a combination of high-throughput screening assessed by predictive biomarkers.

## Figures and Tables

**Table 1:** Chronological report of halophilic bacteria and their molecules with antimicrobial activity in vitro against human pathogens.

Isolation Source	Genus	Antimicrobial Activity	Molecule	Formula	Reference
Saline soil of Kovalam solar salterns India	*Nocardiopsis* sp. AJ1	*E. coli*, *S. aureus*, *P. aeruginosa*, *V. parahaemolyticus*, *A. hydrophila*	Pyrrolo (1,2-A (pyrazine-1,4-dione, hexahydro-3-(2-methylpropyl)-)	C_11_H_18_N_2_O_2_	[40]
			Actinomycin C2	C_63_H_88_N_12_O_16_	
Sfax solar saltern, Tunisia	*Paludifilum halophilum* SMBg3	*E. coli* BW25113, *S. henoxaz* ATCC43972, *P. aeruginosa* ATCC 49189 Gram-positive *M. luteus* LB 14110, *S. aureus* ATCC6538, and *L. ivanovii* BUG 496)	Cyclic lipopeptide:		[49]
			Gramicidin S	C_60_H_92_N_12_O_10_	
			Cyclic dipeptides (CDPs):		
			Cyclo(l-4-OH-Pro-l-Leu)	C_11_H_18_N_2_O_3_	
			Cyclo(l-Tyr-l-Pro)	C_14_H_16_N_2_O_3_	
			Cyclo(l-Phe-l-Pro)	C_14_H_16_N_2_O_2_	
			Cyclo(l-Leu-l-Pro)	C_11_H_18_N_2_O_2_	
Brine and sediments from Manaure solar saltern. La Guajira, Colombia	*Vibrio* sp. A1SM3–36-8	Methicillin-resistant *S. aureus* (MRSA) ATCC BAA-44, *B. subtilis* ATCC 21556	13-*cis*-docosenamide	C_22_H_43_NO	[51]
Salt lake soil, Algerian Sahara. Algeria	*Nocardiopsis* sp. HR-4	*S. aureus* ATCC 25923, Methicillin-Resistant *S. aureus* (MRSA) ATCC 43300, *M. luteus* ATCC 4698, *E. faecalis* ATCC 29212	Angucyclines and angucyclinones:		[41]
			Compound **1**: (−)-8-*O*-methyltetrangomycin	C_20_H_16_O_5_	
			Compound **2**: (−)-7-deoxy-8-*O* methyltetrangomycin	C_20_H_18_ O_5_	
Topsoil saltern in Jeungdo, Jeollanam-do, Republic of Korea	*Nocardiopsis* sp. HYJ128	*Salmonella enterica* ATCC 14028	Borrelidin C	C_28_H_43_NO_7_	[42]
			Borrelidin D	C_28_H_43_NO_7_	
Sediments of mangrove Nizampatnam, Bay of Bengal, Andhra Pradesh, India	*Pseudonocardia endophytica* VUK-10	*B. cereus* (MTCC 430), *S. mutans* (MTCC 497), *S. aureus* (MTCC 3160), *S. epidermis* (MTCC 120), *B. subtilis* (ATCC 6633), *B. megaterium* (NCIM 2187), *E. coli* (ATCC 35218), *P. aeruginosa* (ATCC 9027), *P. vulgaris* (MTCC 7299), *S. marcescens* (MTCC 118), *X. campestris* (MTCC 2286), *X. malvacearum* (NCIM 2954) and *S. typhi* (ATCC 14028)	*N*-(4-aminocyclooctyl)-3,5-dinitrobenzamide	C_15_H_20_N_4_O_5_	[50]
			3-((1H-indol-6-yl) methyl) hexahydropyrrolo [1,2-a] pyrazine-1,4-dione	C_16_H_17_N_3_O_2_	
Soil sample, Xinjiang Province, China	*Streptomonospora alba* YIM 90003	*B. anthracis*, *B. halodurans*, *B. cereus* ATCC 4342, ATCC 13472, *B. subtilis*, *L. monocytogenes*, *E. faecalis*, *S. aureus* and *M. smegmatis*	Streptomonomicin (STM)	C_107_H_160_N_22_O_30_	[48]
Great Barrier Reef (GBR) sponges, Queensland, Australia	*Salinispora* *arenicola*	*M. avium*, *M. leprae*, *M. lepromatosis*, *M. tuberculosis*	Rifamycin B	C_39_H_49_NO_14_	[56]
			Rifamycin S	C_37_H_45_NO_12_	
			Rifamycin W	C_35_H_45_NO_11_	
**Saline soil,** Qaidam Basin, north-west China	*Nocardiopsis terrae* YIM 90022	*S. aureus*, *E. coli* and *B. subtilis*	Quinoloid alkaloid 4-oxo-1,4-dihydroquinoline-3-carboxamide	C_10_H_7_N_2_O_2_	[43]
			*p*-hydroxybenzoic acid	C_7_H_6_O_3_	
			*N*-acetyl-anthranilic acid	C_9_H_9_NO	
			Indole-3-carboxylic acid	C_9_H_7_NO_2_	
			Cyclo (Trp-Gly)	C_13_H_13_N_3_O_2_	
			Cyclo (Leu-Ala)	C_9_H_16_N_2_O_2_	
Condenser water, solar salt works in Thamaraikulam, Kanyakumari district, Tamil Nadu, India	*Bacillus* sp. BS3	*E. coli*, *S. aureus*, *P. aeruginosa* and *S. typhi*	Lipopeptide biosurfactants		[53]
			13-Docosenamide, (Z)	CH_3_(CH_2_)_7_CH=CH(CH_2_)_11_CONH_2_	
			Mannosamine	C_6_H_13_NO_5_.HCl	
			9-Octadecenamide, (Z)	C_18_H_35_NO	
			2-Octanol, 2-methyl-6-methylene	C_12_H_22_O_2_	
			Cylohex-1,4,5-triol-3-one-1-carbo	C_5_H_8_FN_3_	
			2-Butanamine, 2-methyl-	C_5_H_13_N	
			1,2-Ethanediamine, *N*,*N*,*N*′,*N*′-tetramethyl-	C_6_H_16_N_2_	
Hypersaline soil, Xinjiang, China	*Nocardiopsis gilva* YIM 90087	*B. subtilis*, *S. aureus*	*p*-Terphenyl: 6′-Hydroxy-4,2′,3′,4′′-tetramethoxy-p-terphenyl	C_22_H_22_O_5_	[44]
			*p*-Terphenyl derivative: 4,7-bis(4-methoxyphenyl)-6-hydroxy-5-methoxybenzo[d]thiazole	C_22_H_19_NO_4_S	
Solar salt condenser, Thamaraikulam solar saltern, Kanyakumari district, Tamil Nadu, India	*Halomonas salifodinae* MPM-TC	*V. harveyi*, *V. parahaemolyticus*, *P. aeruginosa* and *A. hydrophila*	Perfluorotributylamine	C_12_F_27_N	[54]
			Cyclopentane, 1-butyl-2-ethyl-	C_11_H_22_	
			1,1′-Biphenyl]-3-amine	C_12_H_11_N	
			Pyridine, 4-(phenylmethyl)-	C_12_H_11_N	
			Hexadecane, 2-methyl-	C_17_H_36_	
			Nonadecane	C_19_H_40_	
			Phytol	C_20_H_40_O	
Seashore soil, Bigeum Island, South West coast of South Korea	*Streptomyces hygroscopicus* BDUS 49	*B. subtilis*, *S. aureus*, *E. coli*, *S. typhi*	7-Demethoxy rapamycin	C_50_H_75_NO_12_	[47]
Marine sediment of Mission Bay, San Diego, South California	*Marinispora* sp. NPS12745	*S. aureus* ATCC 29213-MSSA, *S. aureus* ATCC 43300-MRSA, *S. epidermidis* ATCC 700578, *S. epidermidis* ATCC 700582, *S. pneumoniae* ATCC 49619-Penicillin sensitive, *S. pneumoniae* ATCC 51915-Penicillin resistant, *E. faecalis* ATCC 29212-Vancomycin sensitive, *E. faecium* ATCC 700221-Vancomycin resistant, *Haemophilus influenzae* ATCC 49247, *Haemophilus influenzae* ATCC 49766 *E. coli* permeable mutant	Chlorinated bisindole pirroles:		[57]
			Lynamicin A	C_22_H_16_N_3_O_2_Cl_2_	
			Lynamicin B	C_22_H_14_N_3_O_2_Cl_3_Na	
			Lynamicin C	C_20_H_12_N_3_Cl_4_	
			Lynamicin D	C_24_H_18_N_3_O_4_Cl_2_	
			Lynamicin E	C_24_H_19_N_3_O_4_Cl	
Platinum Coast on the Mediterranean Sea, north of Egypt	*Streptomyces* sp. Merv8102	*E. coli* ATCC 10536, *P. aeruginosa* ATCC 10145), *B. subtilis* ATCC 6051, *S. aureus* ATCC 6538 and *M. luteus* ATCC 9341	Essramycin Triazolopyrimidine [1,2,4] Triazolo[1,5-a]pyrimidin-7(4H)-one, 5-methyl-2-(2-oxo-2-phenylethyl)-	C_14_H_12_N_4_O_2_	[58]
Marine sediment, La Jolla, California	*Streptomyces* sp. CNQ-418	Methicillin-resistant *S. aureus* (MRSA)	Marinopyrroles A	C_22_H_12_Cl_4_N_2_O_4_	[59]
			Marinopyrroles B	C_22_H_11_BrCl_4_N_2_O_4_	
Sediment of Bay of Bengal, India	*Streptomyces chibaensis* sp. AUBN1/7	*B. subtilis* ATCC 6633, *B. pumilus* ATCC 19164, *S. aureus* ATCC 29213, *E. coli* ATCC 25922, *P. aeruginosa* ATCC 27853 *P. vulgaris* ATCC 6897	1-Hydroxy-1-norresistomycin	C_21_H_14_O_7_	[45]
Sediment of the Lagoon de Terminos at the Gulf of Mexico	*Streptomyces* B8005*Streptomyces* B4842	*E. coli*, *S. aureus*, *S. viridochromogenes*	Resistomycin 1-Hydroxy-1-norresistomycin	C_21_H_14_O_7_	[60]
			Resistoflavin Resistoflavin methyl ether	C_23_H_18_O_7_	
Marine sediment from Scripps Canyon. La Jolla, California, Pacific Coast, United States	*Streptomyces nodosus* NPS007994	Drug-sensitive and drug-resistant Gram-positive reaction bacteria	Lajollamycin Nitro-tetraene Spiro-β-lactone-γ-lactam	C_36_H_53_N_3_O_10_	[61]
Sediment of Jiaozhou Bay, China	*Actinomadura* sp. M048	*S. aureus*, *B. subtilis*, and *S. viridochromogenes*	Chandrananimycin A Acetamide, *N*-(9-hydroxy-3-oxo-3H-phenoxazin-2-yl)-	C_14_H_10_N_2_O_4_	[62]
			Chandrananimycin B Acetamide, 2-hydroxy-*N*-(3-oxo-3H-phenoxazin-2-yl)-	C_14_H_10_N_2_O_4_	
			Chandrananimycin C 1-Methoxy-3-methyl-1,2,3,4-tetrahydro-5H-pyrido[3,2 a]phenoxazin-5-one	C_17_H_16_N_2_O_3_	
Sandy sediment, coastal site of Mauritius, Indian Ocean	*Streptomyces* sp. B6921	*S. aureus*, *E. coli*, *B. subtilis*, and *S. viridochromogenes*	Fridamycin D	C_31_H_32_O_12_	[46]
			Himalomycin A	C_43_H_52_O_16_	
			Himalomycin B	C_43_H_56_O_16_	
Mucus secreted by the box- fish *Ostracion cubicus*, Israel	*Vibrio parahaemolyticus* B2	*S. aureus*, *S. albus* and *B. subtilis*	Vibrindole A	C_18_H_16_N_2_	[52]

**Table 2:** Chronological report of bacteria with antimicrobial activity in vitro against human pathogens which molecules have not been chemically identified.

Isolation Source	Genus	Antimicrobial Activity	Reference
Khewra Salt Range, Punjab, Pakistan	*Aquisalibacillus elongatus* MB592, *Salinicoccus sesuvii* MB597, and *Halomonas aquamarina* MB598	*B. subtilis*, *B. pumilus*, *E. faecalis*, *B. cereus*, *K. pneumoniae*, *Alcaligenes faecalis*, *P. geniculata*, *E. faecium*	[63]
Hypersaline soils (solonchaks, solonetz and takyr) from Kostanay, Auliekol and Mendykara. Almaty region, Balkhash, Kazakhstan	*Actinomycetes* spp.	*S. aureus* MRSA, *E. coli* (pMG223)	[64]
Marine water, Gujarat, Western India	*Kocuria* sp. strain rsk4	Antibiotic-resistant *S. aureus*	[65]
Crystallizer pond sediments of Ribandar saltern, Goa, India	*Streptomyces radiopugnans*	*S. typhimurium*, *P. vulgaris*, *E. coli*	[66]
	*Streptomyces sporocinereus*	*S. typhimurium*, *P. vulgaris*, *E. coli*	
	*Kocuria palustris*	*S. aureus*	
	*Micromonospora* sp.	*V. cholerae*	
	*Nocardiopsis* sp.	*S. citreus*	
Coastal Solar Saltern, India	*Nonomuraea* sp. JAJ18	Methicillin-Resistant *S. aureus* (MRSA), *B. subtilis* MTCC 441, *K. pneumonia* MTCC 109, *S. typhi* MTCC 733, and *P. vulgaris* MTCC 426	[67]
Sediment of estuarine coastal brackish, Chilika Lake, Khurdha Odisha, India	*Streptomyces chilikensis* RC 1830	*E. coli*, *S. aureus*, *B. cereus* and *S. typhi*	[67]
Mangrove sediment of Visakhapatnam, Andhra Pradesh, India	*Streptomyces* sp.	*S. aureus*, *B. subtilis*, *B. cereus*, *E. coli*, *P. aeruginosa*, *P. vulgaris*	[68]
Mangrove sediment, Nizampatnam, Andhra Pradesh, India	*Pseudonocardia* VUK-10	*S. aureus*, *S. mutans*, *B. subtilis*, *E. coli*, *E. faecalis*, *P. aeruginosa*	[69]
Salt pans Batim and Ribandar, Goa, India	*Bacillus* spp. *Virgibacillus* spp.	*A. baumanii*, *A. hydrophila*, *Citrobacter diversus*, *Citrobacter freundii*, *E. coli* ATCC 25922, *K. pneumoniae*, *Morganella morganii*, *P. mirabilis*, *P.* ATCC 27855, *P.* spp., *S. paratyphi* A, *S. typhi,* *S. typhimurium,* *S. boydii*, *S. flexneri*, *V. cholerae*, Methicillin Resistant *S. aureus* (MRSA), Methicillin Sensitive *S. aureus* (MSSA), *S. aureus* ATCC 25923, *S. citreus*	[70]
Salt pans, Kodiakarai, Tamil Nadu, India	*Streptoverticillium album*	*S. aureus, K. pneumoniae* and *E. coli*	[71]
Nonrhizospheric soil, Saharan regions, south of Algeria	*Actinopolyspora* spp. *A. halophila*, *A. mortivallis*, *A. erythraea*, *A. xinjiangensis*, *A. alba.Nocardiopsis* spp. *N. litoralis*, *N. xinjiangensis* *N. valliformis* and *N. exhalans* *Saccharomonospora* spp. *S. paurometabolica*, *S. halophila* *Streptomonospora* spp. *S. alba*, *S. amylolytica*, *S. flavalba* *Saccharopolyspora* sp.	*B. subtilis*, *S. aureus*, *M. luteus*, *K. pneumoniae*, *L. monocytogenes*	[72]
Crystallizer pond, Madurai, India	*Nocardiopsis* sp. JAJ16	*S. aureus*, *B. subtilis*, *S. typhi*, Methicillin-resistant *S. aureus* (MRSA), *K. pneumoniae*, *Enterobacter* sp. and *P. aeruginosa*	[73]
Bay of Bengal coast of Puducherry and Marakkanam, India	*Streptomyces* sp. VITSVK9	*B. subtilis*, *Escherchia coli*, *K. pneumoniae*, *S. aureus* and *S.* species	[74]
Marine sediment of Marakkanam, Bay of Bengal Coast, Tamil Nadu. India	*Saccharopolyspora salina* VITSDK4	*S. aureus* ATCC 25923, *B. subtilis* ATCC 6633, *E. coli* ATCC 25922, *K. pneumoniae* ATCC 10273	[75]
Marakkanam coast of Tamil Nadu, India	*Streptomyces* sp. VITSDK1	*S. aureus* ATCC 25923, *B. subtilis* ATCC 6633, *E. coli* ATCC 25922, *K. pneumoniae* ATCC 10273	[76]
Salt Lake Hami in Xinjiang, China	*Actinomyces* sp.	*B. subtilis*	[77]
Salt lakes of Bay of Bengal, India	*Actinomyces* sp. *Streptomyces* sp.	*P. aeruginosa*, *B. subtilis*, *S. epidermidis*, *E. coli*	[78]
Water samples Asen fjord in the Trondheim fjord and Steinvikholmen, Norway	*Streptomyces* sp.	Gram-negative and Gram-positive bacteria	[79]
Salt Lake Bardawil, Egypt	*Streptomyces viridiviolaceus*	*E. coli*, *Edwardsiella tarda*, *Corynebacterium michiganese* B-33, *P. solanacearum* B-3212 and *Staphilococcus* spp.	[77]
Soil from salt pan regions of Cuddalore and Parangipettai (Porto-Novo). Tamil Nadu, India	*Streptomyces* sp., *Saccharomonospora* sp.	*E. coli*, *K. pneumoniae*, *P. aeruginosa*, *V. cholerae*, *S. typhi*, *S. aureus*, and *S. dysenteriae*	[80]
Bismarck and Solomon Sea off the coast of Papua New Guinea	*Micromonospora nigra* DSM 43818, *Micromonospora rhodorangea*, *Micromonospora halophytica* DSM 43171	Multidrug-resistant (MDR) Gram-positive pathogens, vancomycin-resistant enterococci (VRE), and methicillin-resistant *S. aureus* (MRSA)	[81]
Marine sediment, Alibag coast, Maharashtra, India	*Actinopolyspora* spp. AH1, *A.halophila*, *A. mortivallis*, *A. iraqiensis*	*S. aureus*, *S. epidermidis*, *B. subtilis*	[82]

Noted: American Type Culture Collection (ATCC); Deutsche Sammlung von Mikroorganismen und Zellkulturen (DSMZ); Multidrug-resistant (MDR); Microbial Type Culture Collection and Gene Bank (MTCC). Microorganisms: *Acinetobacter* (*A.*)*: A. baumanii. Aeromonas* (*A.*)*: A. hydrophila. Alcaligenes* (*A.*)*: A. faecalis. Bacillus* (*B.*)*: B. cereus, B. halodurans, B. megaterium, B. pumilus, B. subtilis. Burkholderia* (*B.*)*: B. metallica. Candida* (*C.*)*: C. albicans. Citrobacter* (*C.*)*: C. diversus, C. freundii. Corynebacterium* (*C.*)*: C. michiganese. Edwardsiella* (*E.*)*: E. tarda. Enterobacter* (*E.*)*: E. aerogenes. Enterococcus* (*E.*)*: E. faecalis, E. faecium,* Vancomycin resistant *Enterococcus faecium* (*VREF*)*,* Vancomycin sensitive *Enterococcus faecalis* (*VSEF*)*,* Vancomycin resistant enterococci (*VRE*)*. Escherichia* (*E.*)*: E. coli. Haemophilus* (*H.*)*: H. influenzae. Klebsiella* (*K.*)*: K. pneumonia. Listeria* (*L.*)*: L. ivanovii, L. monocytogenes. Micrococcus* (*M.*)*: M. luteus. Morganella* (*M.*)*: M. morganii. Mycobacterium* (*M.*)*: M. avium, M. leprae, M. lepromatosis, M. smegmatis, M. tuberculosis. Proteus* (*P.*)*: P. mirabilis, P. vulgaris. Pseudomonas* (*P.*)*: P. aeruginosa, P. geniculata, P. solanacearum. Salmonella* (*S.*)*: S. henoxaz, S. paratyphi, S. typhi, S. typhimurium. Serratia* (*S.*)*: S. marcescens. Shigella* (*S.*)*: S. boydii, S. dysenteriae, S. flexneri. Staphylococcus* (*S.*)*: S. aureus, S. citreus, S. epidermidis,* Antibiotic-resistant *Staphylococcus aureus* (*ARSA*)*,* Methicillin Sensitive *Staphylococcus aureus* (*MSSA*)*,* Methicillin-resistant *Staphylococcus aureus* (*MRSA*)*. Streptococcus* (*S.*)*: S. mutans, S. pneumoniae,* Penicillin resistant *Streptococcus pneumoniae* (*PRSP*)*,* Penicillin sensitive *Streptococcus pneumoniae* (*SPPS*)*. Streptomyces* (*S.*)*: S. viridochromogenes. Vibrio* (*V.*)*: V. cholerae, V. harveyi, V. parahaemolyticus. Xanthomonas* (*X.*)*: X. campestris, X. malvacearum.*

**Table 3:** Halophilic fungi showing antimicrobial activity.

Isolation Source	Species	Antimicrobial Activity	Molecule	Formula	Reference
Abyssal marine sediment. Barents Sea. Arctic Ocean	*Aspergillus protuberus* MUT 3638	*S. aureus*, *K. pneumoniae*, *A. baumanii* and *B. metallica*	Bisvertinolone	C_28_H_33_O_9_	[87]
Solar saltern, Phetchaburi, Thailand	*Aspergillus flavus*, *Aspergillus gracilis*, and *Aspergillus penicillioids*	Antibacterial and antioxidant	Crude extracellular compounds	NR	[102]
Putian saltern of Fujian, China	*Aspergillus flocculosus* PT05-1	*E. aerogenes*, *P. aeruginosa*, and *C. albicans*	Ergosteroids: (22*R*,23*S*)-epoxy-3b,11a,14b,16b-tetrahydr- oxyergosta-5,7-dien-12-one	C_28_H_42_O_6_	[100]
			Pyrrole derivates: 6-(1H-pyrrol-2-yl) hexa-1,3,5-trienyl-4-methoxy-2H-pyran-2-one	C_16_H_15_NO_3_	
Putian saltern of Fujian, China	*Aspergillus terreus* PT06-2	*E. aerogenes*, *P. aeruginosa*, and *C. albicans*	Terremide A	C_21_H_17_N_3_O_5_	[101]
			Terremide B	C_21_H_15_N_3_O_4_	
			Terrelactone A	C_24_H_26_O_8_	
Semiarid saltpans in Botwana	*Aspergillus terreus* Tsp22	*B. megaterium* and *S. aureus*	Crude extracellular compounds	NR	[103]

Abbreviations: Not reported (NR). Microorganisms: *Acinetobacter* (*A.*): *A. baumanii. Bacillus* (*B.*): *B. megaterium. Burkholderia* (*B.*): *B. metallica. Candida* (*C.*): *C. albicans. Enterobacter* (*E.*): *E. aerogenes. Escherchia* (*E.*): *E. coli. Haemophilus* (*H.*): *H. influenzae. Klebsiella* (*K.*): *K. pneumonia. Pseudomonas* (*P.*): *P. aeruginosa. Staphylococcus* (*S.*): *S. aureus*.

**Table 4:** Halophilic bacteria, archaea, and fungi and their relation to cancer treatment.

Anticancer Activity of:	Isolation Source	Halophilic Strain	Cancer Cell Lines	Molecule	Formula	Reference
Bacteria						
Metabolite	Marakkanam saltern and Pichavaram mangroveForest in India	*Bacillus* sp. VITPS16	Cervical carcinoma	Squalene	C_30_H_50_	[116]
				3-Methyl-2-(2-oxopropyl) furan	C_8_H_10_O_2_	
				Methyl hexadeconate	C_17_H_34_O_2_	
	Topsoil saltern in Jeungdo, Jeollanam-do, Republic of Korea	*Nocardiopsis* sp. HYJ128	Stomach and Leukemia carcinoma	Borrelidin C	C_28_H_43_NO_7_	[42]
				Borrelidin D	C_28_H_43_NO_7_	
	Saltern in Incheon in Korea	*Bacillus* sp. KCB14S006	Cervical carcinoma Myeloid leukemia	Iturin F_1_	C_51_H_80_N_12_O_15_Na	[115]
				Iturin F_2_	C_51_H_80_N_12_O_15_Na	
				Iturin A_8_	C_51_H_80_N_12_O_14_Na	
				Iturin A_9_	C_51_H_80_N_12_O_14_Na	
	A saltern on Shinui Island in Korea	*Streptomyces* sp.	Colorectal cancer Gastric cancer	Salternamide A	C_23_H_32_ClNO_5_	[111]
	Salt marsh soil, Alicante, Spain	*Nocardiopsis lucentensis* DSM 44048	Liver cancer Cervical cancer cells	Nocarbenzoxazole G	C_15_H_13_NO_4_	[114]
-	Brine-seawater interface of the Red Sea	12 halophilic marine strains	Breast adenocarcinoma Cervical carcinoma Prostate carcinoma	Crude extract	NR	[108]
-	Deep-sea brine pools of the Red Sea	24 halophilic marine strains	Breast adenocarcinoma Cervical carcinoma Prostate carcinoma	Crude extract	NR	[109]
-	Weihai Solar Saltern in China	*Streptomyces* sp. WH26	Lung adenocarcinoma Liver hepatocellular adenocarcinoma Cervical carcinoma Colorectal cancer	8-*O*-Methyltetrangulol	C_20_H_14_O_4_	[110]
-				Naphthomycin A	C_40_H_46_ClNO_9_	
-	Baicheng salt field, Xingjiang Province, China	*Actinopolyspora erythraea* YIM 90600	Tumor suppressor Programmed Cell Death Protein 4 (Pdcd4)	Actinopolysporins A	C_15_H_28_O_4_	[107]
				Actinopolysporins B	C_16_H_30_O_4_	
				Actinopolysporins C	C_16_H_30_O_2_	
	Weihai Solar Saltern in China	45 moderately halophilic strains	Liver hepatocellular adenocarcinoma	Crude extracts	NR	[106]
Supernatant metabolite	Sambhar Lake in India	*Piscibacillus* sp. C12A1	Breast adenocarcinoma	Crude extract	NR	[118]
	Brine and sediment of the Manaure solar saltern in Colombia	*Vibrio* sp. A1SM3-36-8	Lung adenocarcinoma	13-*cis*-docosenamide	C_22_H_43_NO	[51]
	Different hypersaline lakes in Iran	9 moderately halophilic strains	Umbilical vein endothelial cancer cell	Crude extract	NR	[113]
Biosurfactant	Thamaraikulam solar salt works in India	*Halomonas* sp. BS4	Mammary epithelial carcinoma	1,2-Ethanediamine, *N*,*N*,*N*’,*N*’-tetra	C_6_H_16_N_2_	[119]
				8-Methyl-6-nonenamide	C_10_H_19_NO	
				9-Octadecenamide, (*Z*)	C_18_H_35_NO	
	Solar salt works in India	*Bacillus* sp. BS3	Mammary epithelial carcinoma	13-Docosenamide, (*Z*)	CH_3_(CH_2_)_7_CH=CH(CH_2_)_11_CONH_2_	[53]
				Mannosamine	C_6_H_13_NO_5_·HCl	
				9-Octadecenamide, (*Z*)	C_18_H_35_NO	
				2-Octanol,2-methyl-6-methylene	C_12_H_22_O_2_	
				Cylohex-1,4,5-triol-3-one-1-carbo	C_5_H_8_FN_3_	
				2-Butanamine, 2-methyl-	C_5_H_13_N	
				1,2-Ethanediamine, *N*,*N*,*N*′,*N*′-tetramethyl-	C_6_H_16_N_2_	
Exopolysaccharide	Çamalti saltern area in Turkey	*Halomonas smyrnensis* strain AAD6	Breast adenocarcinoma Lung adenocarcinoma Liver hepatocellular adenocarcinoma Gastric adenocarcinoma	Levan	C_18_H_32_O_16_	[121]
	Sabinar saline wetland in Spain	*Halomonas stenophila* strain B100	Lymphoblastic leukemia	Single acidic exopolysaccharide with glucose, mannose and galactose	NR	[120]
Carotenoid	Industrial tannery wastewater in Iran	*Kocuria* sp. MA-2	Prostate carcinoma	Neurosporene	C_40_H_58_	[122]
Enzyme	Hypersaline soil in Iran	*Halomonas elongata* IBRC-M 10216	Lymphoblastic leukemia Myeloid leukemia	l-asparaginase	C_1377_H_2208_N_382_O_442S17_	[124]
Archaea						
Supernatant metabolite	Aran Bidgol hypersaline lake in Iran	*Halobacterium salinarum* IBRC-M 10715	Prostate carcinoma	Crude extract	NR	[105]
Exopolysaccharide	Urmia Lake in Iran	*Halorubrum* sp. TBZ112	Gastric adenocarcinoma	Monosaccharide composition mainly composed of mannose, glucosamine, galacturonic acid, arabinose, and glucuronic acid	NR	[125]
Carotenoid	Marine solar saltern in eastern China	*Halogeometricum limi* strain RO1-6 *Haloplanus vescus* strain RO5-8	Liver hepatocellular adenocarcinoma	Bacterioruberin	C_50_H_76_O_4_	[127]
	Tunisian solar saltern	*Halobacterium halobium*	Liver hepatocellular adenocarcinoma	Bacterioruberin	C_50_H_76_O_4_	[126]
**Fungi**						
Metabolite	Weihai Solar Saltern in China	*Aspergillus* sp. F1	Lung adenocarcinoma Liver hepatocellular adenocarcinoma Cervical carcinoma Colorectal cancer	Cytochalasin E	C_28_H_33_NO_7_	[128]
				Ergosterol	C_28_H_44_O	
				Rosellichalasin	C_28_H_33_NO_5_	

Abbreviations: Not reported (NR).

**Table 5:** Promising new compounds derived from halophilic microorganisms candidates for preclinical trials.

Compound	Structure	Antibiotic Activity		Anticancer Activity		Reference
		Microorganism	MIC (μM)	Cell Lines	IC_50_ (μM)	
Borrelidin C, D	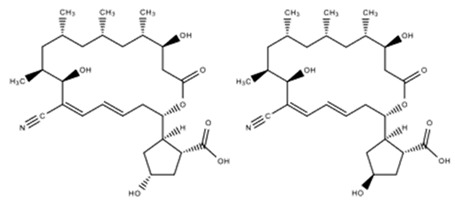	*S. enterica*	16–63	Stomach	5.5	[42]
				Leukemia	5.7	
				Leukemia	6.7	
Angucyclinone: *N*-(4-aminocyclooctyl)-3,5-dinitrobenzamide	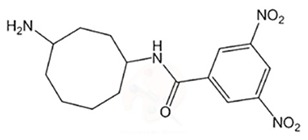	*S. aureus*, *S. epidermis*, *B. subtilis*, *B. megaterium*, *P. aeruginosa*	16	Breast, cervical, ovarian cyst, adenocarcinoma	10 nM	[50]
		*S. mutans*	4			
		*X. malvacearum*, *S. typhi*, *E. coli*	32			
		*B. cereus*	8			
		*C. albicans*	16			
Streptomonomicin STM	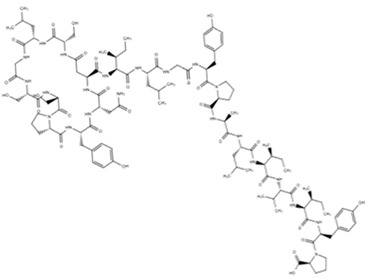	*B. anthracis*	2–4	NR	NR	[48]
		*B. halodurans*	4			
		*B. cereus*	4–7			
		*Bacillus* sp.	7			
		*B. subtilis*	29			
		*L. monocytogenes*	14			
		*E. faecalis*	29			
		*S. aureus*	57			
4-oxo-1,4-dihydroquinoline-3-carboxamide	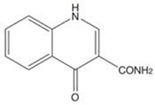	*S. aureus*	64	NR	NR	[43]
		*B. subtilis*	64			
6′-Hydroxy-4,2′,3′,4″-tetramethoxy-p-terphenyl	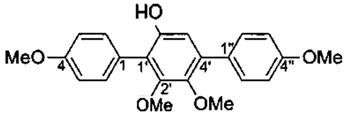	*B. subtilis*	64	NR	NR	[44]
		*C. albicans*	32			
Lynamicin A, B, C, and D	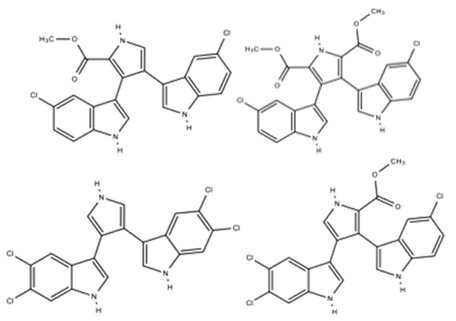	*S. aureus*	1.8–6.2	NR	NR	[57]
		*S. epidermidis*	2.2–9.5			
		*S. pneumoniae*	18–57			
		*E. faecalis*	3.3–19			
		*E. faecium*	4.4–19			
		*H. influenzae*	4.4–38			
		*E. coli*	13–16			
Essramycin	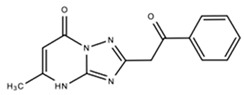	*E. coli*	8	NR	NR	[58]
		*P. aeruginosa*	3.5			
		*B. subtilis*, *S. aureus*	1			
		*M. luteus*	1.5			
Resistomycin 1-hydroxy-1-Norresistomycin	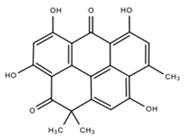	*E. coli*	40	NR	NR	[60]
		*S. aureus*				
		*S. viridochromogenes*				
Resistoflavin methyl ether	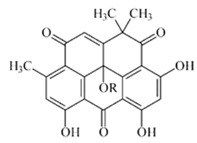 R=Me	*B. subtilis*	3.1	NR	NR	
		*E. coli*, *S. aureus*, *C. albicans*	10			
Lajollamycin	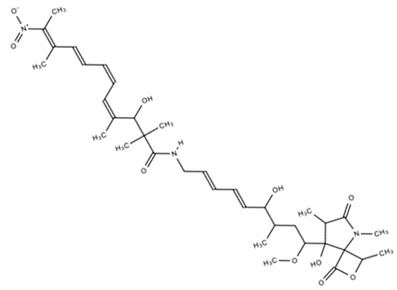	*MSSA*	4	Murine melanoma cell line B16-F10	9.6	[61]
		*MRSA*	5			
		SPPS	2			
		PRSP	1.5			
		VSEF	14			
		VREF	20			
		*E. coli*	12			

Note: Not reported (NR). Microorganisms: *Bacillus* (*B.*)*: B. cereus, B. halodurans, B. megaterium, B. subtilis, B. anthracis. Candida* (*C*.)*: C. albicans. Enterococcus* (*E.*)*: E. faecalis, E. faecium,* Vancomycin resistant *Enterococcus faecium (VREF),* Vancomycin sensitive *Enterococcus faecalis* (*VSEF*)*, Vancomycin resistant enterococci* (*VRE*). *Escherichia* (*E.*)*: E. coli. Haemophilus* (*H.*)*: H. influenzae. Listeria* (*L.*)*: L. monocytogenes. Micrococcus* (*M.*)*: M. luteus. Pseudomonas* (*P.*)*: P. aeruginosa. Salmonella* (*S.*)*: S. typhi, S. enterica. Staphylococcus* (*S.*)*: S. aureus, S. epidermidis, S. mutans*, Methicillin Sensitive *Staphylococcus aureus* (*MSSA*)*, Methicillin-resistant Staphylococcus aureus* (*MRSA*)*. Streptococcus* (*S.*)*: S. pneumoniae*, Penicillin resistant *Streptococcus pneumoniae* (*PRSP*)*,* Penicillin sensitive *Streptococcus pneumoniae* (*SPPS*)*. Streptomyces* (*S.*)*: S. viridochromogenes. Xanthomonas* (*X.*)*: X. malvacearum.*
